# Level of Knowledge and Attitude Toward Acute Abdomen Among the Public: A Nationwide Study

**DOI:** 10.7759/cureus.52416

**Published:** 2024-01-16

**Authors:** Jubran J Al-Faifi, Khalid A Alruwaili, Abdulhakeem H Alkhenizan, Mohammed F Alharbi, Faisal N Alammar

**Affiliations:** 1 Department of Surgery, College of Medicine, Imam Mohammad Ibn Saud Islamic University, Riyadh, SAU; 2 Department of Medicine, College of Medicine, Imam Mohammad Ibn Saud Islamic University, Riyadh, SAU

**Keywords:** saudi arabia, surgical emergencies, genral surgery, emergency care, acute abdominal pain

## Abstract

Introduction

Treatment of patients suffering from acute abdominal pain (AAP) is often challenging due to its wide range of associated illnesses, which could delay or prevent the identification of the correct diagnosis. Multiple diagnoses should be considered, particularly those in life-threatening situations needing urgent intervention.

Aim

This study aimed to assess the level of knowledge among the general population related to acute abdomen and which specialty should be sought for consultation in AAP situations.

Subject and methods

This cross-sectional study was conducted among the general population living in Saudi Arabia. A self-administered questionnaire was sent to the Saudi population through the Google survey. The questionnaire included basic demographic characteristics (i.e., age, gender, etc.), perceived causes, barriers, the most common symptoms of AAP, and a five-item questionnaire to assess the general population's knowledge about AAP.

Results

Of the 2,703 respondents, 68.1% were females, and 42.4% were aged between 18 and 25 years old. The digestive system disorders (esophagus, stomach, intestine, and colon) were the most perceived causes that led to AAP (76.6%). The overall mean knowledge score was 1.35 (SD 1.07) out of five points. Accordingly, nearly all (87.2%) were considered to have poor knowledge, 9.5% were considered to have moderate knowledge, and only 3.3% were considered to have good knowledge. Being younger, being a male, and living in the Central Region were the factors associated with increased knowledge.

Conclusion

There was a lack of knowledge about AAP among the general population in Saudi Arabia. Younger age, gender males, and living in the Central Region were identified as the significant factors of increased knowledge. It is necessary to increase the knowledge of the population regarding acute abdomen. Media awareness campaigns may play a significant role in providing the necessary information to the general public.

## Introduction

Acute abdomen is one of the most serious presentation in patients presenting to the hospital, which needs urgent and proper diagnosis. It is defined as severe abdominal pain lasting for hours to a few days. The pain could be either localized or diffuse throughout the abdomen. The underlying pathology may be intra-abdominal, thoracic, or systemic and may require urgent surgical intervention. Acute abdomen could be caused by infection, inflammation, obstruction, and vascular injury such as pancreatitis, cholecystitis, appendicitis, peritonitis, inflammatory bowel disease, mesenteric ischemia, abdominal aortic aneurysm, and malignancy. It is a time-sensitive condition requiring immediate diagnosis and treatment to avoid complications, including sepsis, necrosis and/or gangrene, fistula, and death. Statistics show that almost 6% of acute abdomen cases missed diagnosis for other diseases. This cross-sectional study aimed to determine the knowledge of physician specialty choices for consultation among acute abdomen patients in Saudi Arabia [1,2].

The exact frequency and prevalence in Saudi Arabia are still unknown. In a study published in the United States, 11% of emergency room (ER) visits were due to abdominal pain during the years 2004, 2009, and 2014 [3]. Another study conducted at the King Fahad Hospital reported that acute appendicitis was the most common cause of AAP (47% of the cases), followed by nonspecific abdominal pain (19%), gall stones disease (11%), and intestinal obstructions [4].

Acute abdominal pain (AAP) needs prompt diagnosis and treatment. An infection, inflammation, vascular blockage, or obstruction are all potential causes of the acute abdomen, and all of them require different treatment. According to a study, some patients do not know about urgent care clinics (UCCs) and go to the ER for everything, which causes pressure on the ER team (only one‑quarter of the studied patients knew about UCCs)[5]. Some of them are urgent and need to be treated fast to avoid long-term complications for the patients and improve their quality of life. some of them could cause death if not investigated and treated promptly, while some could be treated easily if diagnosed early to avoid surgical or more costly intervention. From the doctors perspective, there are some reasons for delayed management of patients with abdominal pain, including late presentation of the patients where some patients may underestimate the symptoms or desire of the patient to consult their own physicians or specific hospitals.

In the outpatient context, abdominal discomfort is a frequent presentation, and it can be challenging to identify the cause. Abdominal discomfort is the primary complaint in 5% of trips to the ER and 1.5% of doctor office visits [6]. Even though there is a higher prevalence of presentation with abdominal symptoms, most of the cases are mild and do not require surgical intervention [7].

A study conducted by Laurell et al. [8] aimed to identify the differential diagnostic difficulties in AAP at the emergency department and during hospitalization. Any patients who complained of AAP for more than six days between 1997 and 2000 were re-evaluated at one year following discharge. There were 2,851 patients with AAP, which showed that the major differential diagnostic problem and diagnosis requiring surgery is related to nonspecific abdominal pain (1,058 patients; 37.2%) and appendicitis (277; 9.7%), which need careful evaluation due to common diagnostic pitfalls. As shown in the study, it is difficult for emergency department physicians to reach specific diagnoses.

Similarly, Toorenvliet [9] investigated the efficacy and safety of standard outpatient re-evaluation within 24 hours for patients who were not admitted to the hospital after emergency department surgical consultation for AAP between June 2005 and July 2006, resulting in a sample size of 500 patients. In 30% (148 patients) of them, the diagnosis was different from the initial diagnosis, 17% (85 patients) had a change in management, 4% (20 patients) were admitted for surgery, and only 1.2% (six patients) had a delay in diagnosis and management. Lastly, according to the information collected, they reported Standard Outpatient Re-evaluation as safe and effective.

It can be challenging to evaluate an emergency department patient with AAP [10]. Several factors can obscure the presentation, thus delaying or preventing the correct diagnosis and resulting in negative patient outcomes. Clinicians must consider multiple diagnoses, particularly those that are life-threatening and require prompt intervention to reduce morbidity and mortality. This article will go over the general information about abdominal pain and the clinical approach by reviewing the history and physical examination.

Only about one-third of GPs used the Rome criteria to diagnose irritable bowel syndrome (IBS) regularly. In most primary care settings, IBS is a diagnosis of exclusion. This has implications for the specialty training of general practitioners and calls into question the applicability of IBS guidelines in daily care, which advocate for an early, positive, symptom-based diagnosis [11].

There is no precise number of cases of acute abdomen in Saudi Arabia. Still, in the United States, using data from 1999 to 2008 by The Centers for Disease Control and Prevention, statistics show that 7% to 10% of emergency department visits were due to abdominal pain [8]. There are many different causes of acute abdomen, including appendicitis, diverticulitis, pancreatitis, cholecystitis, and vascular emergencies, which make it essential to choose the correct physician for a quick and proper diagnosis [12].

Lakhoo et al. [13] published a study aimed to investigate the prevalence and characteristics of abdominal pain in the United States. They reported that 24,929 patients filled out the survey, and around 10,000 patients experienced abdominal pain. Also, 84.5% of cases were transferred to primary care physicians, 39.2% to gastroenterologists, 18.6% to nurse practitioners or physician assistants, 8.3% to obstetricians or gynecologists, 7.1% to general surgeons, and 3.2% to rheumatologists. However, two of five patients did not seek help for their symptoms, and some of them were not diagnosed or treated [13]. Our research focuses on measuring the level of knowledge and attitude related to acute abdomen to estimate the level of awareness of choosing the correct treating physician when patients have symptoms of acute abdomen.

## Materials and methods

This cross-sectional study was conducted among the Saudi general population. The estimated sample size of 1,000 to 2,000 participants for each administrative area (total of 15,000 to 20,000) was collected using the convenience sampling method. All participants of different nationalities, older than 18 years, and not working as health practitioners were included in this study, while respondents were excluded if they were healthcare practitioners, not living in Saudi Arabia, below the age of 18 years, and did not agree to participate. A self-administered questionnaire was distributed to the targeted subjects using an online link that was distributed among the public using different social media platforms using the snowball sampling technique as each participant was asked not to share the link with other participants. At the start of the questionnaire, participants were asked if they had been diagnosed with or were having symptoms of abdominal pain, and those who denied this were excluded. The questionnaire was composed of sociodemographic characteristics (i.e., age, gender, etc.), perceived causes of abdominal pain, barriers to early presentation, the most common symptoms of AAP, and a five-item questionnaire to measure the general population’s knowledge about AAP. This project was approved by the Institutional Review Board (IRB) of Imam Muhammad Ibn Saud Islamic University, with an IRB approval project #453/2023.

Questionnaire criteria

The knowledge of acute abdomen was assessed using a five-item questionnaire, with the correct answer for each question identified and coded as 1 and the incorrect answer coded as 0. The total knowledge score was calculated by adding all five items. The higher the score, the higher the knowledge about acute abdomen. The level of knowledge was then classified into three categories: a score of 0 to 2 points was considered poor knowledge, a score of 3 points was considered moderate, and a score of 4 to 5 points was considered good knowledge.

Statistical analysis

The data were analyzed using the software program Statistical Packages for Software Sciences (SPSS) Version 26 (IBM Corp., Armonk, NY, USA). Descriptive statistics were presented as numbers and percentages (%) for all categorical variables, while continuous variables were computed and summarized as mean and standard deviation. The total knowledge score was compared with the basic demographic characteristics of participants using the Mann-Whitney Z-test and the Kruskal Wallis H-test. Normality tests were performed using the Shapiro-Wilk test and the Kolmogorov-Smirnov test. The total knowledge score followed the non-normal distribution. Therefore, non-parametric tests were applied. Values were considered significant with a p-value of less than 0.05.

## Results

In total, 2,703 participants filled out the survey. The basic demographic characteristics of participants are given in Table 1. Overall, 42.4% were between 18 and 25 years of age, with females being dominant (68.1%). Respondents who lived in the Northern Region constituted 36.8%. Participants who had a personal or family medical history of severe abdominal pain constituted 69.7%.

**Table 1:** Basic demographic characteristics of participants (n=2,703)

Study variables	N (%)
Age group	
18–25 years	1,145 (42.4%)
26–30 years	363 (13.4%)
31–40 years	467 (17.3%)
41–50 years	497 (18.4%)
51–60 years	200 (07.4%)
>60 years	31 (01.1%)
Gender	
Male	862 (31.9%)
Female	1841 (68.1%)
Region	
Central Region	889 (32.9%)
Northern Region	996 (36.8%)
Southern Region	223 (08.3%)
Eastern Region	345 (12.8%)
Western Region	250 (09.2%)
Personal or family medical history of severe abdominal pain	
Yes	1,883 (69.7%)
No	820 (30.3%)

**Figure 1:**
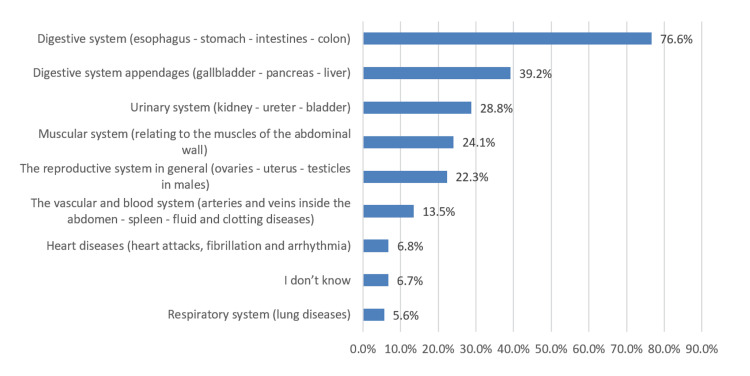
Perceived cause or source of a problem that leads to AAP

As shown in Figure 1, the most common perceived causes of a problem leading to AAP were digestive problems (76.6%), followed by digestive system appendages (39.2%) and urinary system problems (28.8%).

**Figure 2:**
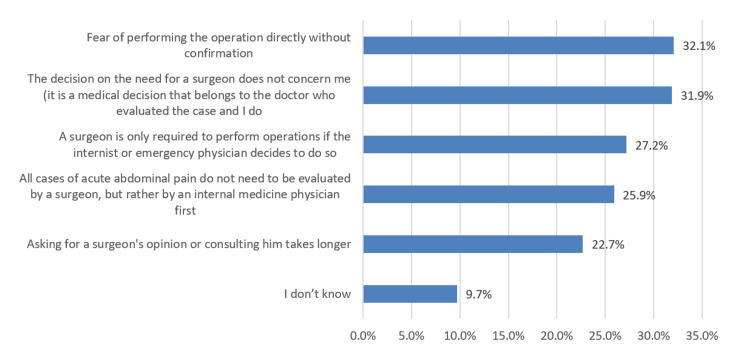
Reason for not consulting a surgeon when there is a case of acute abdominal pain

As shown in Figure 2, the most common reasons for not consulting a surgeon when there is a case of AAP were the fear of performing the operation without confirmation (32.1%) and the decision on the need for surgery to be a medical doctor’s concern (31.9%).

**Figure 3:**
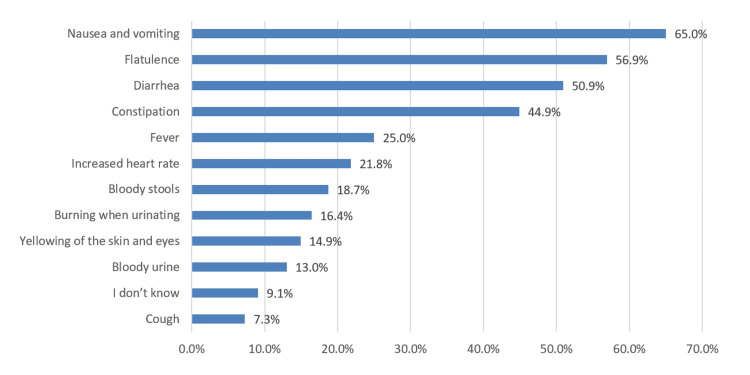
Most common symptoms associated with acute abdominal pain

As shown in Figure 3, the most common symptoms associated with AAP were nausea and vomiting (65%), followed by flatulence (56.9%) and diarrhea (50.9%).

**Figure 4:**
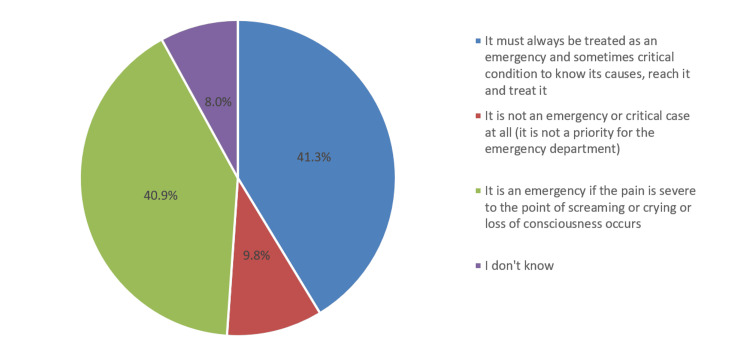
Belief and point of view regarding the condition of acute abdominal pain

As shown in Figure 4, 41.3% of the respondents were of the opinion that acute abdominal must always be treated as an emergency and critical condition.

Regarding the assessment of knowledge about AAP (Table 2), it was observed that only 29.9% were correct about the correct meaning of acute pain, which is “applied to a person who complains of abdominal pain, but only at more severe levels.” Regarding respondents’ opinions in cases of severe abdominal pain that requires medical care, only 11.5% were correct that the patients should be brought directly to the emergency department. When asked about who is the specialist doctor for cases of AAP, most of them (69.9%) indicated the internal medicine doctor, while only 7.3% indicated a general surgeon. According to respondents’ point of view of acute abdominal cases, 41.3% indicated that it should be treated as an emergency where urgent medical care is needed. In addition, 44.6% believed that AAP may require emergency surgery. Based on the above statements, the overall mean knowledge score was 1.35 (SD 1.07), with poor, moderate, and good knowledge levels accounting for 87.2%, 9.5%, and 3.3%, respectively.

**Table 2:** Assessment of participants' knowledge about AAP (n=2,703) †Variable with multiple response answers. *Indicates correct answer. AAP, acute abdominal pain

Statement	N (%)
Knowledge about the correct meaning of AAP.^†^	
I believe that AAP is only applied to a person who screams or cries due to the severity of the pain.	1099 (40.7%)
I believe that AAP applies to a person who complains of any abdominal pain, regardless of the level or duration of time.	744 (27.5%)
I believe that AAP applies to a person with known chronic digestive diseases who suffers from an unusual and new attack pain.	918 (34.0%)
I believe that AAP applies to a person who complains of bouts of abdominal pain that recur at intervals.	676 (25.0%)
I believe that AAP applies to a person who complains of abdominal pain, but only at more severe levels.*	809 (29.9%)
I do not know the meaning of AAP or when to use the term APP.	350 (12.9%)
In the past, or in your opinion, in cases of severe abdominal pain that requires medical care, Who should review?	
Primary care center (general practitioner)	376 (13.9%)
Family medicine clinic (family medicine consultant) or (pediatrics consultant) depending on the patient’s age	298 (11.0%)
Emergency department in the hospital (the emergency physician performs the evaluation, and the internist must be consulted first or make a follow-up appointment)	1039 (38.4%)
Emergency department in the hospital (the emergency doctor evaluates and consults the surgical doctor first)*	312 (11.5%)
Booking in the internal medicine clinic for diseases of the digestive system	504 (18.6%)
Booking at the surgery clinic	27 (01.0%)
I do not know exactly what the appropriate option is	147 (05.4%)
In cases of AAP, and from your point of view, which of the specialist doctors do you think is the most worthy of your trust after evaluating the patient and conducting the examination?	
Internal medicine doctor	1890 (69.9%)
General surgery doctor*	197 (07.3%)
Family medicine doctor	117 (04.3%)
Urologist	68 (02.5%)
Emergency doctor	157 (05.8%)
General practitioner	109 (04.0%)
I ’cannot decide	165 (06.1%)
What do you know about AAP	
It must always be treated as an emergency and sometimes critical condition to know its causes*	1116 (41.3%)
It is not an emergency or critical case at all (it is not a priority for the emergency department)	264 (09.8%)
It is an emergency if the pain is severe to the point of screaming or crying or loss of consciousness	1106 (40.9%)
I ’do not know	217 (08.0%)
Do you think that cases of severe abdominal pain may require emergency surgery?	
Yes*	1205 (44.6%)
No	640 (23.7%)
I ’do not know	858 (31.7%)
Total knowledge score (mean ± SD)	1.35 ± 1.07
Level of knowledge	
Poor	2,358 (87.2%)
Moderate	257 (09.5%)
Good	88 (03.3%)

When measuring the differences in the score of knowledge and the basic demographic characteristics of participants (Table 3), it was observed that a higher knowledge score was more associated with being younger (Z=3.279; p=0.001), being male (Z=1.331; p=0.039), and living in the Central Region (H=31.823; p<0.001).

**Table 3:** Differences in the score of knowledge in relation to the basic demographic characteristics of participants (n=2,703) §P-value has been calculated using the Mann-Whitney Z-test. ‡P-value has been calculated using Kruskal-Wallis H-test. **Significant at p<0.05 level.

Factor	Knowledge score (5), mean ± SD	Z-test	P-value^§^
Age group			
≤30 years	1.41 ± 1.10	3.279	0.001**
>30 years	1.26 ± 1.02		
Gender			
Male	1.41 ± 1.14	1.331	0.039**
Female	1.32 ± 1.03		
Region			
Central Region	1.45 ± 1.02	31.823	<0.001**,^‡^
Northern Region	1.23 ± 1.08		
Southern Region	1.39 ± 1.12		
Eastern Region	1.39 ± 1.11		
Western Region	1.33 ± 1.07		
Personal or family medical history of severe abdominal pain			
Yes	1.37 ± 1.07	1.842	0.065
No	1.29 ± 1.08		

In post hoc analysis (Table 4), it was revealed that there was a significant difference in the score of knowledge between participants living in the Central Region and participants living in the Northern Region (p<0.001).

**Table 4:** Multiple mean differences of knowledge score in relation to region (n=2703) Post hoc test has been conducted using the Dunn Bonferroni test. *The mean difference is significant at the 0.05 level.

Region (I)	Region (J)	Mean difference (I-J)	Std. error	P-value	95% confidence interval	
					Lower bound	Upper bound
Central Region	Northern Region	0.22139^*^	0.04930	0.000	0.0829	0.3599
	Southern Region	0.06767	0.08002	1.000	-0.1571	0.2925
	Eastern Region	0.06491	0.06777	1.000	-0.1255	0.2553
	Western Region	0.12532	0.07648	1.000	-0.0896	0.3402
Northern Region	Central Region	-0.22139^*^	0.04930	0.000	-0.3599	-0.0829
	Southern Region	-0.15372	0.07915	0.522	-0.3761	0.0686
	Eastern Region	-0.15648	0.06674	0.191	-0.3440	0.0310
	Western Region	-0.09607	0.07558	1.000	-0.3084	0.1162
Southern Region	Central Region	-0.06767	0.08002	1.000	-0.2925	0.1571
	Northern Region	0.15372	0.07915	0.522	-0.0686	0.3761
	Eastern Region	-0.00276	0.09180	1.000	-0.2607	0.2551
	Western Region	0.05765	0.09841	1.000	-0.2188	0.3341
Eastern Region	Central Region	-0.06491	0.06777	1.000	-0.2553	0.1255
	Northern Region	0.15648	0.06674	0.191	-0.0310	0.3440
	Southern Region	0.00276	0.09180	1.000	-0.2551	0.2607
	Western Region	0.06041	0.08874	1.000	-0.1889	0.3097
Western Region	Central Region	-0.12532	0.07648	1.000	-0.3402	0.0896
	Northern Region	0.09607	0.07558	1.000	-0.1162	0.3084
	Southern Region	-0.05765	0.09841	1.000	-0.3341	0.2188
	Eastern Region	-0.06041	0.08874	1.000	-0.3097	0.1889

## Discussion

The present study evaluated the general population’s knowledge regarding acute abdomen and determined which specialty should be sought for consultation in AAP situations. The findings of this study revealed that the general public knowledge about AAP was inadequate. According to our criteria, 87.2% had poor knowledge, 9.5% had moderate, and only 3.3% had good knowledge (mean score: 1.35; SD 1.47, out of five points). This is the first study in Saudi Arabia that investigated the level of understanding of the general public regarding AAP, which is an important subject given that this disease is considered an emergency event. Moreover, comparing our results to medical students’ knowledge about AAP [14], it was observed that medical students were more than equipped with knowledge than the general population. According to reports, 58% of the medical students were classified as having a good level of awareness, while the rest (42%) had poor awareness (mean score: 23.4; SD 9.4, out of 32 points). Generally, the knowledge of the public was assumed to be lower than that of the medical population. Hence, healthcare providers, including medical students, may also provide support to educate the public about the basic facts of acute abdomen, including the most appropriate specialist for this type of disease to bridge knowledge gaps.

Data from our study suggest that younger age groups, male gender, and respondents living in the Central Region tended to be more knowledgeable than the rest of the groups. This is almost mirrored by the study published by Alamrani et al. [14], in which age, gender, educational year, and nationality showed a significant relationship with the level of awareness. However, in Iran [15], they documented no significant relationship between the field of specialty and the mean knowledge score of the medical residents, but they found significant differences between the mean percentages of correct answers of the various professional groups (51.2% medical residents vs. 36.1% nurses, p<0.001).

The lack of understanding among our population stemmed from their specific knowledge about the basic facts of AAP. For instance, many of our subjects incorrectly identified the meaning of AAP, wherein 40% selected that “it is a condition applied to a person who screams or cries due to the severity of the pain” (40.7%), when in fact, “it is a condition given to a person who complains of abdominal pain at higher severe levels” (29.9%). Our population correctly recognized that the emergency department should be sought for cases of AAP. Still, they incorrectly tagged internists to be consulted after initial evaluation instead of a surgeon. Most of our respondents have chosen internal medicine doctors as the most appropriate specialists to conduct examinations for AAP patients (69.9%) instead of a general surgeon (7.3%). Furthermore, our respondents cited “fear of conducting the operation without doing confirmation” as the most common barrier to consulting a surgeon (32.1%). Other mentioned barriers were as follows: “the decision is not their concern” (31.9%), “performing operation is based on internists or ER physicians’ decisions” (27.2%), and “AAP cases do not need an evaluation of the surgeons” (22.7%). In the USA [13], patients who sought care for abdominal pain mostly preferred primary care physicians (84.5%), followed by gastroenterologists (39.2%), nurse practitioners or physician assistants (18.6%), and rheumatologists (3.2%). Only 7.1% had visited the general surgeons.

According to our subjects’ point of view, digestive system disorders (esophagus, stomach, intestines, and colon) were one of the major causes of AAP (76.6%), followed by digestive system appendages (gallbladder, pancreas, and liver) (39.2%), urinary systems (kidney, ureter, bladder) (28.8%), and muscular system (24.1%). Other causes, but with fewer ratings, were heart diseases (6.8%) and respiratory problems (5.6%). Furthermore, we noted that nausea and vomiting (65%), flatulence (56.9%), diarrhea (50.9%), and constipation (44.9%) were the commonest symptoms of AAP. Several studies documented appendicitis as AAP’s most common presenting complaint [12,16-19]. However, in India [20] and Italy [21], the most common cause of acute abdomen was nonspecific abdominal pain, accounting for 14% and 31.5%, respectively. On the other hand, in a prospective cohort study conducted in Tanzania [22], malignancies (36%), intestinal obstruction (6%), and peptic ulcer disorder (5%) were the most common final diagnoses among patients with abdominal pain presenting to the emergency department. In addition, 41.3% of our subjects believed that acute abdominal should always be treated as an emergency whose causes should be known, while 40.9% believed that pain should be severe in order to be classified as an emergency case.

Limitations

The study on abdominal symptoms encounters several challenges due to the subjective nature of the topic and the lack of a consistent definition in the literature. The severity of a symptom becomes challenging to define without a clear understanding of the underlying pathology. Given the broad spectrum of potential pathologies associated with abdominal discomfort, patient enrollment, and their medical history, particularly any prior experiences with AAP or related pathologies, are likely to significantly influence their perception of the underlying problem and the perceived significance of the symptoms.

Moreover, the study faces the issue of recall bias, as participants may struggle to accurately recall and report symptoms associated with AAP. The reliability of the collected data may be compromised by the inherent limitations of human memory. Additionally, depending on the method of questionnaire distribution, the study might be susceptible to bias associated with the online platform. Variability in participant demographics, access to healthcare, and willingness to disclose symptoms online could introduce confounding factors that impact the generalizability of the findings.

## Conclusions

The knowledge of the Saudi general population regarding AAP was relatively low. However, younger males living in the Central Region may exhibit a better knowledge of AAP than the rest of the population. Further, in the case of AAP, internists were perceived to be more trustworthy doctors instead of general surgeons. Fear of performing the operation without the necessary confirmation of the disease was the most prominent barrier to consulting a surgeon. This study provides evidence regarding the lack of knowledge about AAP. However, due to the scarcity of this study discipline, further research is needed to extract more data about the general population’s knowledge of AAP.
